# A Microfluidic Paper-Based Device for Monitoring Urease Activity in Saliva

**DOI:** 10.3390/bios15010048

**Published:** 2025-01-15

**Authors:** Francisca T. S. M. Ferreira, António O. S. S. Rangel, Raquel B. R. Mesquita

**Affiliations:** CBQF—Centro de Biotecnologia e Química Fina, Laboratório Associado, Universidade Católica Portuguesa, Escola Superior de Biotecnologia, Rua Diogo Botelho 1327, 4169-005 Porto, Portugal; fferreira@ucp.pt (F.T.S.M.F.); arangel@ucp.pt (A.O.S.S.R.)

**Keywords:** kinetic determination, gas-diffusion membrane, point-of-care, saliva sample

## Abstract

Chronic Kidney Disease (CKD) is a disorder that affects over 10% of the global population, and that would benefit from innovative methodologies that would provide early detection. Since it has been reported that there are high levels of urease in CKD patients’ saliva, this sample is a promising non-invasive alternative to blood for CKD detection and monitoring. This work introduces a novel 3D µPAD for quantifying urease activity in saliva in a range of 0.041–0.750 U/mL, with limits of detection and quantification of 0.012 and 0.041 U/mL, respectively. The device uses the urease in the sample to convert urea into ammonia, causing a colorimetric change in the bromothymol blue. The accuracy of the developed device was evaluated by comparing the measurements of several saliva samples (#13) obtained with the μPAD and with a commercially available kit. Stability studies were also performed to assess its functionality as a point-of-care methodology, and the device was stable for 4 months when stored in a vacuum. After the sample placement, it could be scanned within 40 min without providing significantly different results. The developed device quantifies urease activity in saliva within 30 min, providing a simple, portable, lab-free alternative to existing methodologies.

## 1. Introduction

The 17 Sustainable Development Goals (SDGs) created in 2015 by the United Nations aimed to encourage the partnership between all developed and developing countries to move towards a peaceful and prosperous future for the planet and its civilization [1]. However, with the recent COVID-19 pandemic, the necessity of uniting for the common well-being of the global population became even more imperative. Point-of-care diagnosis devices have also received more attention due to the COVID-19 pandemic and COVID self-testing.

Microfluidic paper-based analytical devices, µPADs, are portable, disposable, and affordable devices capable of performing rapid analytical determinations without requiring complex equipment or specialized training [2,3,4]. These devices usually comprise a hydrophilic zone, where the reaction occurs, and a hydrophobic zone that limits the reaction area [4]. Using paper as the hydrophilic platform for the reaction is the preferred material due to its affordability, high availability in different porosities and treatments, and easy storage and transport [3,4]. Furthermore, the microchannels present in the cellulose fibres allow the flow of analytes and reagents simply by capillary force, without the need for external driving devices [2,4,5]. The hydrophobic area, on the other hand, can be achieved by physically blocking the paper’s pores or chemically modifying the paper fibbers [3,5]. Some of the most commonly reported materials and techniques are wax printing, inkjet printing, photolithography, cutting, and polydimethylsiloxane plotting [2,3,5,6]. Furthermore, µPADs can also be fabricated with 2D or 3D structures [2,5,6]. The 2D devices have a simpler assembly/fabrication process because they are composed of only one layer of paper, consequently relying only on the lateral flow on that layer of paper [2,6]. On the other hand, 3D µPADs are composed of several layers of paper, which can be achieved by stacking or folding and provide the ability to perform more complex analysis [4,5,6]. In these devices, the several layers of paper allow lateral and vertical flow through the layers [2,5]. This can be advantageous since it gives a higher control and flow speed and facilitates the possibility of performing several chemical reactions in a specific order [2,5]. In terms of detection methods, several techniques have already been reported; however, the most commonly used in µPADs is colorimetric detection [4,7]. Since it allows a simpler signal readout that can be performed either by observing the colour (qualitative to semi-quantitative result) or scanning the device and calculating the intensity of the colour using an image processing software (quantitative result) [4,7]. Because every technique has advantages and limitations, the fabrication, assembly, and readout choices should be carefully thought out according to the device’s objective [2,6].

Chronic Kidney Disease (CKD) is a condition that affects over 10% of the world’s population and is characterized by kidney malfunction, which leads to inadequate blood filtration and build-up of harmful metabolic toxins and waste in the body [2,8,9,10]. Early detection is crucial for slowing disease progression, preventing complications, and preserving patients’ quality of life, especially since currently there is no cure for CKD [2,8,11]. This need is especially pressing in economically vulnerable countries with limited healthcare access, where the prevalence is higher, and undiagnosed CKD can be life-threatening [2,8]. Although blood is the standardized sample for evaluating renal function, its collection process is inconvenient, painful, time-consuming, and invasive [9,10,12]. Moreover, individuals with CKD face an elevated risk of contracting blood-borne illnesses, which elevates the importance of using alternative biological samples of non-invasive collection [13]. Saliva emerges as a viable alternative sample due to its easy, quick, painless, and non-invasive collection method [2]. Furthermore, several studies reported that the urea levels in saliva CKD patients are significantly higher than the ones of healthy individuals [9,13,14]. With the increase in urea in saliva, a modification of the oral microbiota is expected to occur, particularly the proliferation of urease-producing microorganisms [9,10]. This urease enzyme then converts the high levels of urea into ammonia, which gives the patients the characteristic ammonia breath [10,15,16].

In this work, a novel 3D structured microfluidic paper-based device was developed to determine urease activity in saliva samples as a potential aid in diagnosing and monitoring CKD. This determination relies on the enzymatic reaction involving urease, the conversion of urea to gaseous ammonia, which consequently diffuses through a hydrophobic membrane and generates a colorimetric change in the pH indicator bromothymol blue (BTB). This device provides a more straightforward, portable, lab equipment-free, point-of-care alternative to commercially available kits that normally require specific lab equipment and extra steps such as sample pre-treatment [17]. To the best of our knowledge, only one other paper-based device for determining urease activity has been reported, but with an application to soil samples [18].

## 2. Material and Methods

### 2.1. Reagents and Solutions

All solutions used in this work were prepared with analytical grade chemicals and Milli-Q water (resistivity > 18 MΩ·cm, Millipore, Burlington, MA, USA).

A 0.2 M phosphate-buffer solution was prepared by dissolving 4.5 g of K_2_HPO_4_·3H_2_O (Merck, Darmstadt, Germany) in 100 mL of water, and the pH was adjusted to 7. This solution was stored at 2–8 °C in the refrigerator.

The urease enzyme (from *Canavalia ensiformis* (Jack Bean), Sigma-Aldrich, St. Louis, MI, USA) stock solution of 100 U/mL was prepared by dissolving 5.0 mg of the lyophilized powder in 2 mL of phosphate buffer and was stored at −20 °C. A 10-fold dilution was prepared weekly, and standards in the range of 0.05–0.75 U/mL were prepared daily in synthetic saliva.

Synthetic saliva was prepared according to Batista [19] with the following composition: [KCl] = 2.24 g/L; [KH_2_PO_4_] = 0.54 g/L; [CaCl_2_·2H_2_O] = 77.7 mg/L; [MgCl_2_] = 19.4 mg/L; [HEPES] = 4.77 g/L; [Bovine Serum Albumin] = 2.70 g/L [19].

A standard stock solution of 8.4 mM of urea (Sigma-Aldrich, St. Louis, MO, USA) was prepared monthly by dissolving 10 mg of the previously dried solid (overnight at 100 °C) in 20 mL of water.

A 2.5 M sodium hydroxide stock solution was prepared by dissolving 10 g of the solid pellets (Panreac, Barcelone, Spain) in 100 mL of water. A twofold dilution was prepared weekly to a final concentration of 1.25 M.

The colour reagent, 4 mM bromothymol blue indicator (BTB) solution, was prepared by dissolving 25 mg of BTB powder (Merck, Darmstadt, Germany) in 10 mL of ethanol (Panreac, 99.8% (*v*/*v*) Barcelone, Spain). A dilution to 2 mM was prepared and the pH adjusted to 6.5. with NaOH 0.1 M [2].

### 2.2. Design of the Developed µPAD

**Figure 1:**
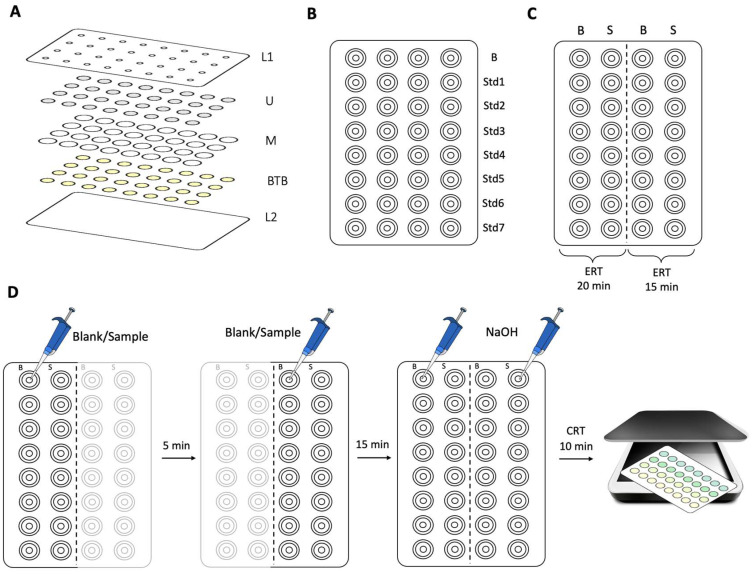
Schematic representation of the developed µPAD’s (**A**) assembly structure; L1, top sheet of the laminating pouch; U, urea layer (15 μL per disc); M, hydrophobic membrane layer; BTB, bromothymol blue layer (15 μL per disc); L2, bottom sheet of the laminating pouch; (**B**) calibration curve structure; B, blank; Std, standard; (**C**) structure for the sample and blank placement; B, blank; S, sample; ERT, enzymatic reaction time; (**D**) determination procedure; CRT, colour reaction time.

In this work, the assembly of the µPAD consists of placing 32 detection units, in 4 columns and 8 lines distribution (Figure 1), inside a plastic laminating pouch (125 microns, A6 size, Leitz, Stuttgart, Germany). These detection units were aligned under the 3 mm diameter sample insertion holes (laser cutting machine, FDA, Model 3040) of the top sheet of the laminating pouch (L1 in Figure 1A).

Each detection unit is composed of 3 layers: the first layer, the urea layer (U in Figure 1A); the middle layer (M in Figure 1A), the hydrophobic polyvinylidene fluoride (PVDF) membrane (1.27 cm diameter, Durapore, 0.45 µm porosity, HVHP09050, Merck, Darmstadt, Germany); and the bottom layer, the BTB layer (BTB in Figure 1A).

The paper discs on the top layer (U in Figure 1A) consisted of Whatman filter paper Grade 4 (9.5 mm diameter), loaded with 15 µL of 8.4 M urea and dried for 20 min in a 50 °C oven. The discs on the bottom layer (BTB in Figure 1A), consisted of Whatman filter paper Grade 1 (9.5 mm diameter), loaded with 15 µL of 2 mM BTB (pH = 6.5) and dried in a 50 °C oven for 10 min. The oven temperature and time ensure the drying of the paper without over-drying.

The plastic pouch with the aligned discs was then laminated (United Office—ULG 300 B1) to melt the plastic around each detection unit, creating a hydrophobic seal between the 32 units.

### 2.3. Determination Procedure

This work aimed to quantify the urease activity by combining the enzymatic reaction of urease with a colorimetric reaction of BTB with the formed product from the enzymatic reaction. The product of the enzymatic urease reaction is ammonia nitrogen, then hydroxide was added to quantitively convert it to gaseous ammonia which diffuses through a hydrophobic membrane. After the diffusion process, the gaseous ammonia alters the pH of the BTB solution causing a colour change. To eliminate the interference of the ammonium in the sample, the urease activity determination was attained by a ΔA calculated from two enzymatic reaction times. The enzymatic reaction, set to occur first, is stopped with the loading of the hydroxide at 15 and 20 min reaction times and then the colour change registered enabling the calculation of A_20min_ and A_15min_ to be used for the ΔA calculation.

To accomplish this in the preparation of the calibration curve (Figure 1B), two devices were prepared: one for a 15 min enzymatic reaction time (ERT) and the other for 20 min ERT, both reaction times followed by the colorimetric reaction involving the ammonium. So, 10 μL of blank and a set of standards were placed on the devices and, after the corresponding ERT, 4 μL of NaOH 1.25 M was loaded into the devices in order to promote the conversion of NH_4_^+^ to NH_3_. The ammonia diffuses through the hydrophobic membrane (M in Figure 1A) to reach the BTB layer (BTB in Figure 1A) causing the colour change. The urease concentration relates to its activity and correlates to subtracting the measurements obtained with the two ERTs, 20 and 15 min. This procedure also enables to account for the NH_4_^+^ already present in the sample, avoiding misleading results with the produced NH_4_^+^ by enzyme activity.

To accomplish the urease activity determination in a sample, a device was prepared and divided into two sections (Figure 1C), one for each ERT. A volume of 10 μL of synthetic saliva (Blank) was loaded into #8 detection units (as illustrated in Figure 1C) together with 10 μL of sample loaded into #8 detection units each (Figure 1C). After 5 min, the same process was carried out on the right side of the μPAD (Figure 1C). Then, after the device has rested for 15 min, 4 μL of NaOH 1.25 M is placed in all the #32 detection units.

To avoid the loss of the gaseous ammonia, the sample insertion holes were covered with adhesive tape immediately after the NaOH was completely absorbed. The scanning of the device is performed on the BTB side (opposite to the sample/standard insertion) 10 min after loading the NaOH, corresponding to the colour reaction time (CRT) (Figure 1D).

The colour change, from yellow to green, is registered by scanning the device and treating the acquired image with an image processing software, ImageJ (Version 1.52q, National Institutes of Health, Bethesda, MD, USA). The intensity counts are obtained applying a red filter to the scanned images as the complementary colour. To measure the intensity of the pixels in each detection area, a circular selection (200 × 200 pixels) was used in the Image J software to adjust the coloured discs better. The values of the colour intensity were converted to absorbance: A = log_10_(I_B_/I_S_), where I_B_ is the intensity of the blank signal and I_S_ is the intensity of the standards/sample signal. To ensure good repeatability, at least four readings of the blank and each of the samples/standards were performed, and outliers were discarded when necessary (relative standard deviations above 10% but ensuring a minimum of three readings).

To establish the calibration curve, the absorbance signal was calculated for each standard as follows: ΔA = A_20min_ − A_15min_, in which ΔA is the variation in absorbance, A_20min_ is the absorbance of the standard with a 20 min ERT, and A_15min_ is the absorbance of the standard with a 15 min ERT. A linear relationship was established between ΔA and the urease concentration in the range of 0.05–0.75 U/mL.

### 2.4. Saliva Samples

Saliva samples from healthy volunteers were collected with their informed consent. The sampling procedure consisted of placing a 5 × 5 cm sterile gauze (Wells) in the mouth for approximately 2 min. Then, the gauze was placed in a 5 mL sterile syringe and squeezed into a 5 mL plastic tube to recover the saliva from the gauze. The samples were spiked with 5 µL of a urease solution of 10 U/mL in 500 µL of sample and analyzed on the same day of the collection.

### 2.5. Comparison Method—Validation Procedure

The accuracy of the developed device was assessed by analyzing saliva samples with the developed µPAD and comparing the results obtained with a commercially available Urease Activity Assay Kit (MAK120, Sigma-Aldrich). For this comparison, #13 saliva samples were spiked with 0.1 U/mL urease.

## 3. Results and Discussion

The development of the urease activity μPAD was based on a previously developed device for urea determination using the enzymatic reaction of urease [2]. In that determination, urea was converted into ammonium by urease, and then ammonium was converted into ammonia by adding hydroxide. The formed ammonia diffused through a hydrophobic membrane to a pH indicator layer, causing a colour change. In the end, the described device was composed of four layers: a top layer consisting of an empty paper disc; a second layer of a paper disc with urease; a third layer corresponding to the hydrophobic membrane; and a bottom layer with the pH indicator paper disc. The top layer consists of an empty paper disc intended to be a physical protective barrier of the urease enzyme layer.

A similar structure was used to perform the urease determination, replacing the urease with a urea standard of 2 mM on the second paper layer. An initial calibration curve was obtained loading urease standards ranging from 10 to 150 U/mL (slope about 3.1 × 10^−4^ mL/U).

### 3.1. Number of Layers

**Figure 2:**
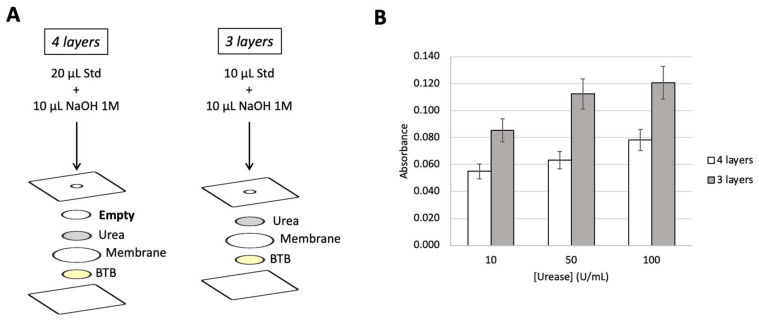
(**A**) Schematic representation of both studied device structures and (**B**) resulting absorbance values obtained from placing urease standards of 10, 50, and 100 U/mL in each studied structure; Std, standard; BTB, bromothymol blue. The error bars correspond to a 10% deviation.

The first optimization study aimed to evaluate the need for the top empty layer, and two devices were prepared, one with the empty paper disc and another without (shown in Figure 2A). Because the absorption capacity of the μPAD decreases when one layer of paper discs is removed, the volume of sample placed in the 3 layers’ μPAD was reduced to half (10 μL) of the 20 μL used in the 4 layers’ μPAD.

It was observed (Figure 2B) that, even with a lower amount of standard, the three-layer structure provided not only higher values of absorbance for each standard (>50%) but also significantly higher overall sensitivity (calibration curve slope higher > 40%). This can be explained as in the four-layer assembly, despite the higher volume, part of the urease gets dispersed/retained in the top empty layer, never reaching the urea layer and consequently never producing ammonia. Thus, there is less ammonia to diffuse through the hydrophobic membrane and reach the BTB layer for colour change, resulting in lower absorbance values and lower sensitivity. Therefore, the three-layer structure was chosen.

### 3.2. Urea Concentration

**Figure 3:**
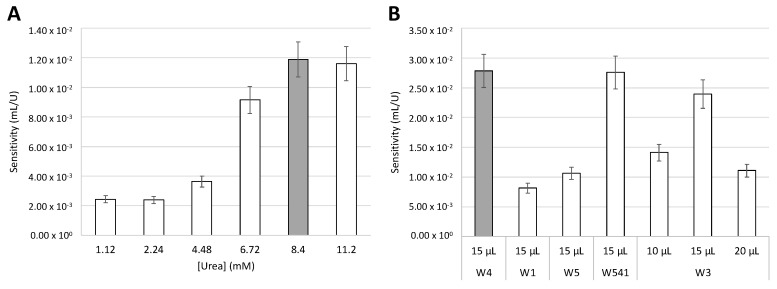
Influence of the (**A**) urea concentration (mM) and (**B**) filter paper type (and correspondent sample volume) on the sensitivity of the calibration curve; W4, Whatman Grade 4 filter paper; W1, Whatman Grade 1 filter paper; W5, Whatman Grade 5 filter paper; W541, Whatman Grade 541 filter paper; W3, Whatman Grade 3 filter paper; the grey bars correspond to the chosen condition. The error bars correspond to a 10% deviation.

Next, the influence of urea concentration on the calibration curve sensitivity was evaluated. Standard urea solutions ranging from 1.12 to 11.2 mM were prepared and loaded in the first paper layer of the developed devices (U in Figure 1A). Then, calibration curves with urease standards (volume of 15 µL) were established. The results (Figure 3A) showed that when using concentrations of 1.12 and 2.24 mM, there was no significant difference (relative deviation of the slopes < 10%) in the sensitivity of the calibration curve. However, when the urea concentration was increased to 8.4 mM, there was an increase in sensitivity of over 380%. The results also showed no significant difference between using 8.4 and 11.2 mM of urea (relative deviation of the slopes < 10%), and, therefore, 8.4 mM was the concentration of urea chosen for the remaining studies.

### 3.3. Type of Paper

The type of paper used was the next optimization study performed since it can significantly interfere with the sensitivity of the device. A variety of filter papers were studied: Whatman Grade 4 (W4), Whatman Grade 1 (W1), Whatman Grade 3 (W3), Whatman Grade 5 (W5), and Whatman Grade 541 (W541). These vary in paper porosity: 2.5 µm (W5), 6 µm (W3), 11 µm (W1), and 20–25 µm (W4 and W541); and in paper treatment: Qualitative (W1, W3, W4, W5) and Hardened Ashless (W541). For this study, a device was prepared with each type of paper and 15 µL of urease standards was placed to obtain a calibration curve. Because W3 has a higher thickness and, therefore, a higher absorption capacity, the standard volumes of 10 and 20 µL when using W3 were included in this study. The sensitivities of the calibration curves were compared (Figure 3B), and the papers that provided a higher calibration curve sensitivity were W4 and W541.

Because there was no significant difference between the sensitivities provided by these two types of paper, and since W541 is more expensive, W4 was the filter paper chosen for the developed device.

### 3.4. Enzymatic Reaction Time

As previously mentioned, the urease quantification device is based on the corresponding enzymatic conversion of urea into ammonia nitrogen. For the quantification, the ammonium present must then be converted to ammonia with the addition of hydroxide, which stops the urease reaction. Because enzymatic reactions are usually highly time-dependent, the next study was performed to assess the influence of different enzymatic reaction times (ERT). Periods of 10, 15, and 20 min between loading the standard and the NaOH were tested, for colour reaction times between 5 and 20 min (ESM Figure S1). It was possible to observe that the increase in ERT from 10 to 15 min always resulted in an increase in the sensitivity (calibration curve slope increase > 30%) for all the tested colour reaction times 5, 10, and 15 min. However, when the ERT was increased to 20 min, the sensitivity of the calibration curve was not statistically different from the sensitivity at 15 min, even showing a slight decrease for both tested colour reaction times (average—19%). This could be explained due to the extended time in contact with air and consequent evaporation affecting the necessary downward vertical flow within the device. As the highest sensitivity was obtained with an ERT of 15 min and colour reaction of 15 min, these were the chosen intervals.

### 3.5. Sodium Hydroxide

**Figure 4:**
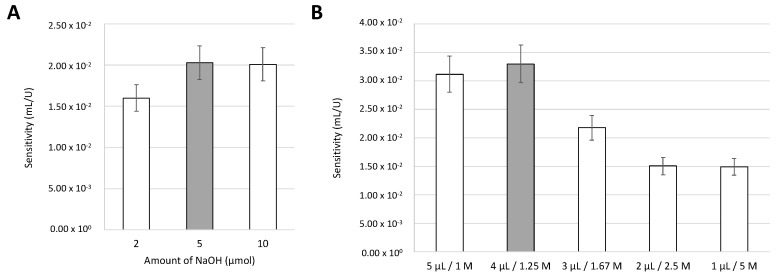
Influence of the (**A**) amount of NaOH (µmol) and (**B**) influence of different proportions of volume/concentration of NaOH, maintaining a final amount of 5 µmol; the grey bars correspond to the chosen condition. The error bars correspond to a 10% deviation.

Then, the influence of the amount of NaOH on the calibration curve was assessed. The amount of NaOH ranging from 2 to 10 µmol was studied (Figure 4A), and it was possible to conclude that the highest sensitivity of the calibration curve was obtained with amounts of 5 and 10 µmol of NaOH. Since there was no significant difference in the sensitivity between these two amounts, 5 µmol was chosen. Then, maintaining the final amount of 5 µmol NaOH, different proportions of volume and concentration were tested (Figure 4B).

The results showed that the proportions that provided the higher sensitivity of the calibration curve were 5 µL of 1 M and 4 µL of 1.25 M. However, because the volume of 4 µL showed an easier absorption into the device, the chosen optimal condition was 4 µL of NaOH 1.25 M.

### 3.6. Matrix Influence

Because the developed device aimed to quantify urease in saliva samples, evaluating the potential interference from the saliva matrix composition was important. Two devices were prepared, and calibration curves were established using two sets of standards, one set of standards prepared in phosphate buffer (recommended by the manufacturer for the stability of the enzyme), and another set prepared in synthetic saliva [19]. The sensitivity of the calibration curves obtained was compared and it was possible to observe that there was an enormous increase (over six-fold) in the sensitivity when using standards prepared in the synthetic saliva. This can happen due to the presence of several compounds that can act as buffer in the reaction, favouring the enzyme activity, and consequently increasing the sensitivity of the device. The final decision was to continue the optimization studies using standards prepared in synthetic saliva.

### 3.7. Kinetic Determination

As mentioned before, the quantification of urease in the developed µPAD is based on the enzymatic conversion of urea to ammonia nitrogen, diffusion and consequent change in the BTB colour (pH indicator). Saliva samples have already a considerable amount of ammonium [2,10,20]. This should not present an interference since this determination is dependent on the kinetic properties of the enzyme. The idea was to perform the measurements at two different ERTs and calculate the variation in the absorbance signal obtained. If the sample contained urease, the signal was expected to increase with the increase in the time allowed for the enzymatic reaction (ERT), since the enzyme would convert more urea to ammonia nitrogen. On the other hand, the ammonium that could already be present in the sample would provide the same signal in both ERTs and therefore be suppressed when calculating the ΔA.

To test this hypothesis, devices were prepared, and two periods of time between standard and NaOH placement (ERTs) were established to calculate the ΔA: absorbance calculation between 10 and 15 min and absorbance calculation between 15 and 20 min (ESM Table S1). The choice of these ERTs is justified by the results obtained in previous tests (Section 3.4) always using the optimum time of 15 min. A couple of urease standards were loaded and the variation in the absorbance (ΔA) values between the two ERTs calculated: (i) ΔA = A_15min_ − A_10min_, where A_15min_ is the absorbance of one standard with a 15 min ERT, and A_10min_ is the absorbance of the same standard with a 10 min ERT; (ii) ΔA = A_20min_ − A_15min_, where A_20min_ is the absorbance of one standard with a 20 min ERT, and A_15min_ is the absorbance of the same standard with a 15 min ERT (ESM Table S1). To further assess the effectiveness of the hypothesis, one sample was also loaded in the same conditions and spiked with ammonium and with urease. Comparing the absorbance signals of the sample and the sample spiked with NH_4_^+^, no significant difference was observed, indicating that the presence of NH_4_^+^ in the sample did not influence the quantification when the signal obtained is the ΔA. On the other hand, when comparing the signal of the sample and the sample spiked with urease, the signal from the urease-spiked sample was significantly higher, as expected. This validates our hypothesis that the kinetic determination allows the quantification of urease and suppresses the influence of NH_X_ already present in the samples.

#### Enzymatic Reaction Kinetics

The interval for the ΔA calculation was further optimized by testing different combinations: 10–15min, 15–20 min, and 10–20 min (ESM Figure S2). It was possible to conclude that 15–20 min was the optimal interval since it provided the highest sensitivity when compared to the intervals (10–15 min RD = −47% and 10–20 min RD = −87%).

### 3.8. Features

The main features of the developed μPAD, such as the dynamic range, the limit of detection and quantification, and the repeatability, both intraday and interday, are summarized in Table 1.

The limit of detection (LOD) and the limit of quantification (LOQ) were calculated as the concentration corresponding to three times (LOD) and ten times (LOQ) the standard deviation of the calibration curves intercept (*n* = 4), according to IUPAC recommendations [21]. The intraday repeatability of the developed device was assessed by calculating the relative standard deviation (RSD) of four replicas of a sample on the same day and same device. The interday repeatability of the developed device was assessed by calculating the relative standard deviation (RSD) of four calibration curve slopes on consecutive days.

### 3.9. Stability Studies

Stability studies were performed to evaluate the performance and robustness of the developed µPAD, not only when stored (before the sample insertion), but also the stability of the coloured product.

The storage stability was evaluated by preparing devices and storing them for different periods under two different atmospheric conditions: in contact with air and in vacuum. The storage devices were wrapped in aluminum foil to be protected from light, and the ones in contact with air were placed inside a clear plastic zip lock bag, while the ones in vacuum atmosphere were placed in a clear vacuum bag (16 × 20 cm, Lacor, Bergara, Spain).

The vacuum atmosphere was achieved using a vacuum packaging machine (MINI/120-ST ECO, Henkovac, ’s-Hertogenbosch, The Netherlands). The devices were stored at room temperature (approximately 21 °C).

**Figure 5:**
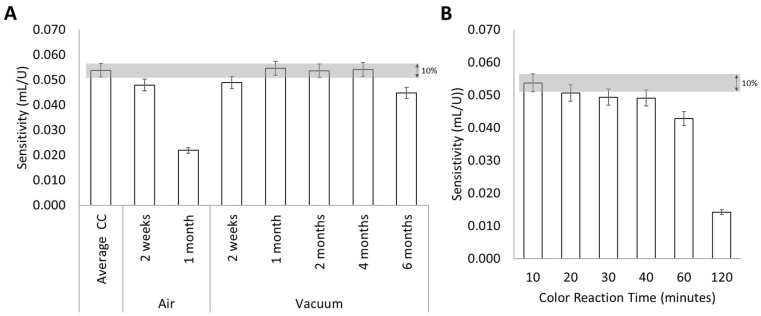
Stability assessment by comparison of the sensitivity of the calibration curves: (**A**) storage stability of the developed device under different atmospheric conditions and periods of time; the grey area represent the 10% deviation of an average calibration curve slope; the error bars correspond to a 5% deviation; (**B**) stability of the coloured product formed on the device under different colour reaction times (CRT); the grey area represent the 10% deviation of a calibration curve slope obtained with a colour reaction time of 10 min. The error bars correspond to a 5% deviation.

After each tested period, a set of urease standards was loaded into the stored μPADs and freshly prepared ones, and the calibration curve slopes were compared by calculating the relative deviation (RD) considering that <10%, there were no differences. After analyzing the results (Figure 5A) it was possible to conclude that the storage in the air atmosphere was not able to maintain the sensitivity of the device in any of the periods tested (RD > 10%). When the µPADs were stored in a vacuum atmosphere, it was possible to observe that the devices were stable (RD < 10%) for at least 4 months.

To assess the stability of the coloured product formed on the developed µPADs, a calibration curve was prepared, and the evolution of the colour in the devices was registered with a scanner at several colour reaction times (CRTs) from the 10 min (used up until this study) to 120 min. CRTs lower than 10 min were not studied since 10 min was the minimum time required for the device to fully absorb the NaOH, to place the adhesive tape and then perform the scan. The results (Figure 5B) showed that the coloured product on developed µPAD was stable for at least 40 min since there was no significant decrease (RD < 10%) in the sensitivity of the obtained calibration curve.

### 3.10. Validation

To evaluate the accuracy of the urease quantification by the developed µPAD, 13 spiked saliva samples were tested on the device, and the results obtained were compared with the measurements from a commercially available urease activity determination kit (Table 2). A linear relationship was established (ESM Figure S3) between the two sets of results, and the following equation was obtained: [Urease]_µPAD_ = 0.993 (±0.067) × [Urease]_Kit_ + 0.003(±0.012). Since the slope was not statistically different from 1 and the intercept was not statistically different from 0, it was possible to conclude that there were no statistically significant differences between the two sets of results.

## 4. Conclusions

In this work, a novel microfluidic paper-based device was developed for the determination of urease activity in saliva samples. Considering both the enzymatic reaction (20 min) and the colorimetric reaction (10 min), this determination is accomplished within 30 min and could be potentially used as an aiding tool in the diagnosis and monitoring of CKD. The urease activity determination was performed in the range of 0.041–0.750 U/mL, with limits of detection and quantification of 0.012 U/mL and 0.041 U/mL, respectively.

The developed μPAD is composed of three layers of stacked filter paper/membrane discs, resulting in a 3D structure that uses both lateral and vertical flow through the layers. The analytical determination within is based on the enzymatic reaction of urease that converts urea into ammonia nitrogen, and on the colorimetric detection of the BTB pH indicator. The gas-diffusion hydrophobic membrane included in the device, not only enhances the selectivity and efficiency of the developed method, but it also eliminates potential interferences from the sample matrix without requiring sample pre-treatment. This was supported by the successful validation of the device with 13 saliva samples from healthy individuals.

To further ensure the robustness of the developed device, stability studies were performed, taking into consideration the storage conditions and the stability of the colour formed on the device after use. The results showed that the developed μPAD would maintain its sensitivity for at least 4 months when stored and protected from light in a vacuum atmosphere. After placing the sample and NaOH on the device, the colour formed can be captured by scanning the μPAD up to 40 min without any significant sensitivity decrease. Additionally, to considerably reduce the common drawback of manually assembled devices (low reproducibility due to potential shifting of discs during assembly and lamination), the developed methodology incorporates the possibility of performing multiple replicates and removing outliers if needed.

To conclude, the developed μPAD is an innovative device that provides a simple, easy-to-use alternative to the classical techniques available for this determination, in addition to being portable, lab equipment-free, and disposable via incineration, offering environmental friendliness and simplifying biological sample handling. According to our search, only one other paper-based device for urease activity determination as been reported [18], but with an application to soil samples. To the best of our knowledge, there are no other reports of paper-based devices for the determination of urease activity in human saliva.

## Figures and Tables

**Table 1:** Features of the developed μPAD for urease determination; LOD, limit of detection; LOQ, limit of quantification; RSD, relative standard deviation.

Dynamic Range (U/mL)	Calibration Curve ^a^ ΔA = S × [Urease] + b	LOD ^a^ (U/mL)	LOQ ^a^ (U/mL)	Repeatability, RSD	
				Sample (Intraday) ^a^	Slope (Interday) ^a^
0.041–0.750	ΔA = 0.0531 (±0.0018) + 0.0142 (±0.0002) R^2^ = 0.9969 (±0.0023)	0.012	0.041	4%	3%

^a^ *n* = 4.

**Table 2:** Analysis of saliva samples with the developed μPADs and comparison of the results obtained with the measurements obtained with the commercially available urease determination kit; RE, relative error.

Sample ID	[Urease]_Kit_ (U/mL)	[Urease]_µPAD_ (U/mL)	RE (%)
1	0.125	0.130	4.2
2	0.140	0.145	3.8
3	0.105	0.095	−9.4
4	0.198	0.189	−4.5
5	0.110	0.116	5.1
6	0.179	0.187	3.9
7	0.234	0.220	−5.9
8	0.114	0.122	7.0
9	0.124	0.133	7.5
10	0.165	0.178	7.6
11	0.363	0.372	2.7
12	0.155	0.141	−8.8
13	0.084	0.092	9.2

## Data Availability

Data are contained within the article or Supplementary Material.

## Supplementary Materials

The following supporting information can be downloaded at: https://www.mdpi.com/article/10.3390/bios15010048/s1, ESM Figure S1: Study of the influence of the Enzymatic Reaction Time (10, 15, 20 min) and of the Colour Reaction Time (5, 10, 15 and 20 min) in the calibration curve slope; ESM Figure S2: Influence of the enzymatic reaction time interval (in minutes) in the sensitivity of the developed device; the error bars correspond to a 10% deviation; ESM Figure S3: Linear relationship between the results obtained with the developed µPAD ([Urease]µPAD) and with the commercially available kit ([Urease]Kit); ESM Table S1: Preliminary assessment of the effectiveness of the kinetic determination; variation in the absorbance reading in different time intervals for two urease standards (0.1 and 0.5 U/mL) and one sample without any addition (Sample), with addition of NH_4_^+^ (Sample_NH_4_^+^), and with addition of Urease (Sample_Urease).
