# Supplementary material for: A Microfluidic Paper-Based Device for Monitoring Urease Activity in Saliva
Source: Biosensors (Basel). 2025 Jan 15;15(1):48. doi: 10.3390/bios15010048 (PMC11763955; doi:10.3390/bios15010048)
Supplement: Supplementary file 1 [file biosensors-15-00048-s001.zip › biosensors-3361781-supplementary.pdf]

ELECTRONIC SUPPLEMENTARY MATERIAL

A microfluidic paper-based device for monitoring the urease activity in saliva

Francisca T. S. M. Ferreira, António O. S. S. Rangel, Raquel B. R. Mesquita*

Universidade Católica Portuguesa, CBQF - Centro de Biotecnologia e Química Fina – Laboratório Associado, Escola Superior de Biotecnologia, Rua Diogo Botelho 1327, 4169-005, Porto, Portugal

*rmesquita@ucp.pt

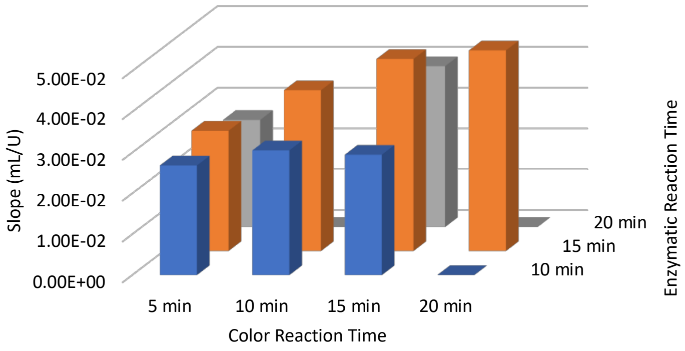

ESM Figure S1. Study of the influence of the Enzymatic Reaction Time (10, 15, 20 minutes) and of the Color Reaction Time (5, 10, 15 and 20 minutes) in the calibration curve slope.

ESM Table S1. Preliminary assessment of the effectiveness of the kinetic determination; variation of the absorbance reading in different time intervals for two urease standards (0.1 and 0.5 U/mL) and one sample without any addition (Sample), with addition of NH_4^+(Sample_NH_4^+), and with addition of Urease (Sample_Urease).

	ΔA	
	[10-15] min	[15-20] min
0.1 U/mL	0.009	0.005
0.5 U/mL	0.022	0.037
Sample	0.003	0.002
Sample_NH_4^+	0.003	0.004
Sample_Urease	0.041	0.035

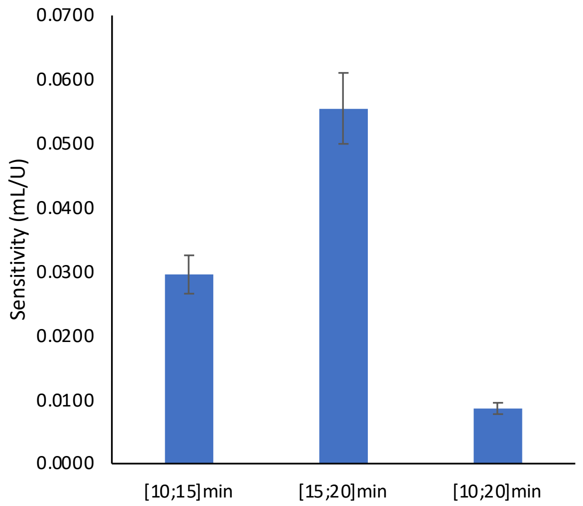

ESM Figure S2. Influence of the enzymatic reaction time interval (in minutes) in the sensitivity of the developed device; the error bars correspond to a 10% deviation.

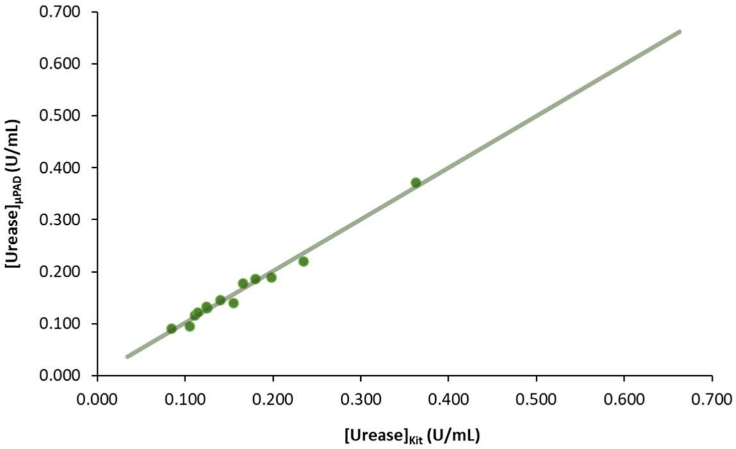

ESM Figure S3. Linear relationship between the results obtained with the developed μPAD ($[Urease]_{\mu PAD}$) and with the commercially available kit ($[Urease]_{Kit}$).
